# Spatially organizing biochemistry: choosing a strategy to translate synthetic biology to the factory

**DOI:** 10.1038/s41598-018-26399-0

**Published:** 2018-05-29

**Authors:** Christopher M. Jakobson, Danielle Tullman-Ercek, Niall M. Mangan

**Affiliations:** 1 Department of Chemical and Systems Biology, Stanford University School of Medicine, Stanford, CA 94305 USA; 2 Department of Chemical and Biological Engineering, Northwestern University, Evanston, IL 60208 USA; 3 Department of Engineering Science and Applied Mathematics, Northwestern University, Evanston, IL 60208 USA

## Abstract

Natural biochemical systems are ubiquitously organized both in space and time. Engineering the spatial organization of biochemistry has emerged as a key theme of synthetic biology, with numerous technologies promising improved biosynthetic pathway performance. One strategy, however, may produce disparate results for different biosynthetic pathways. We use a spatially resolved kinetic model to explore this fundamental design choice in systems and synthetic biology. We predict that two example biosynthetic pathways have distinct optimal organization strategies that vary based on pathway-dependent and cell-extrinsic factors. Moreover, we demonstrate that the optimal design varies as a function of kinetic and biophysical properties, as well as culture conditions. Our results suggest that organizing biosynthesis has the potential to substantially improve performance, but that choosing the appropriate strategy is key. The flexible design-space analysis we propose can be adapted to diverse biosynthetic pathways, and lays a foundation to rationally choose organization strategies for biosynthesis.

## Introduction

Synthetic biology traces its origins to the discovery of type II restriction endonucleases^1,2^. These enzymes allowed the controlled assembly of novel genes, plasmids, and other nucleic acids, and precipitated the rapid spread of molecular cloning technology. Since then, synthetic biology has sought to exploit, adapt, and extend biological systems to benefit society by creating pharmaceuticals, fuels, gene therapies, drug delivery platforms, probiotics, and more. Metabolic engineering, the use of microbes to produce small and large molecules of commercial and scientific interest, has been a particular focus. To this end, synthetic biologists have developed methods to control transcription and translation, knock out native genes to route metabolic flux down desired channels, integrate multiple chemical and physical inputs to make decisions inside microbial cells, and make wholesale changes to the genomes of organisms in high throughput.

**Figure 1:**
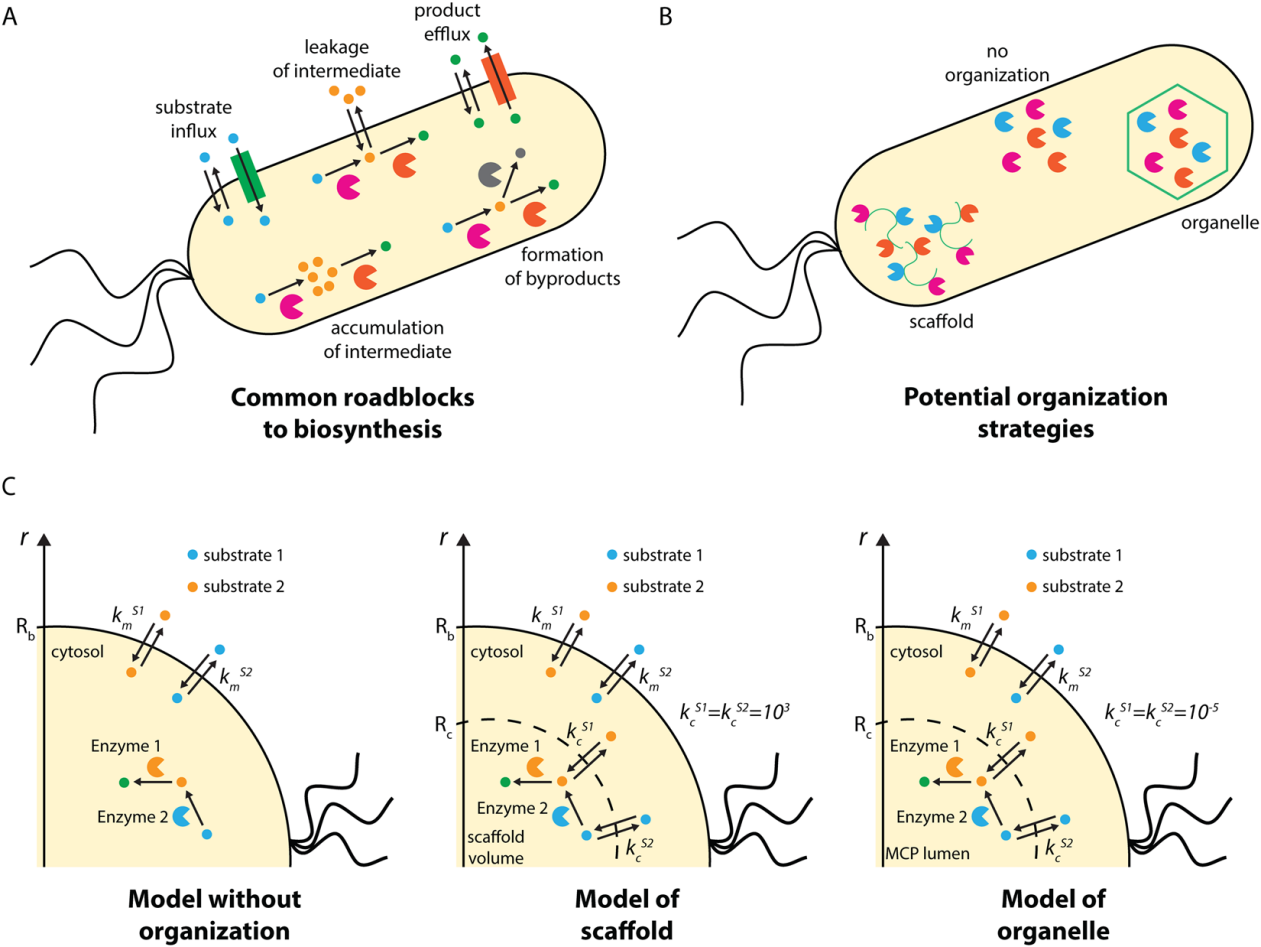
(**A**) Roadblocks commonly facing heterologous biosynthesis. (**B**) Potential organization strategies to alleviate these roadblocks. (**C**) Schematics of our models of (left) a pathway without organization, (middle) a pathway organized on a scaffold, and (right) a pathway organized in an organelle.

Despite technological advances in metabolic engineering and synthetic biology, there have been relatively few examples of the commercially successful, industrial scale production of non-native chemicals by microbes^3^. Historically, acetone-butanol-ethanol (ABE) fermentations using *Clostridium acetobutylicum* have met with large-scale success, as has the production of penicillin from *Penicillium* species, but producing biomolecules not native to the host has presented a much greater challenge. Notable examples include artemisinic acid^4^, as well as farnesene, 1,3-propanediol, and 1,4-butanediol^3^, but, by and large, the heterologous production of chemicals at industrially viable titers has remained elusive. This is most often due to one (or more) of five ubiquitous roadblocks to biosynthesis: cellular toxicity due to accumulation of intermediates of the biosynthetic pathway; the loss of flux to undesired byproducts; difficulties sustaining sufficient substrate influx; leakage and loss of intermediates into the culture medium; and trapping of product in the host cell due to inadequate efflux (Fig. 1A).

A new wave of synthetic biology technologies aims to address these key issues using a diverse array of strategies, while also preparing to deploy engineered microbes widely and safely. These cutting-edge approaches include cell-free approaches to protein and small molecule synthesis^5–11^, dynamic control of metabolite concentrations^12^ and of transcription and translation at the RNA level^13,14^, robust approaches to biocontainment^15^, sensing of diverse small molecules^16^, establishing consortia of synergistic microbes^17,18^, and discovering enzymes facilitating previously unknown catalyses^19,20^. Broadly speaking, these strategies address a key missing capability in the synthetic biological toolkit: the ability to control precisely when and where chemical reactions take place^21–23^.

A new paradigm in synthetic biology technologies focuses on this challenge: spatiotemporal organizational of biochemical processes. Organisms in all domains of life exert fine control over when and where biochemical reactions occur, be they responsible for metabolism, information transfer, or cell replication. This kind of organization remains conspicuously absent from most engineered systems. Here, we will analyze one class of these strategies: the spatial organization of metabolism within cells (Fig. 1B)^24^. There are a wide variety of natural methods for spatial organization^25^. Eukarya discretize their biochemistry into highly chemically distinct subcellular compartments and into enzyme complexes such as polyketide synthases^26^ and other metabolons^27^. Bacteria, too, are now understood to organize their metabolism in a variety of ways, including using protein-based carboxysomes^28^, microcompartments^29^, and encapsulins^30^. 1,2-propanediol utilization (Pdu) microcompartments, for instance, are protein-bound organelles of approximately 150 nm in diameter. The enclosing protein shell consists of trimeric, pentameric, and hexameric protein tiles with central pores that permit the passage of small molecules in and out of the organelles, but which prohibit the passage of enzymes and other proteins. Building on foundational microbiological understanding of these systems^29,31–34^, we and others have demonstrated control of the formation^35^, protein content^36–39^, catalytic activity^40^, and transport properties^41^ of these organelles. Due to their relative simplicity and ease of manipulation, these various protein-based compartments make excellent model systems for exploring the role of spatial organization on metabolism. It is thought that they function both by protecting the cellular contents from toxic intermediates^42^ and by increasing the local concentration of the kinetically relevant intermediate inside the organelle^43^.

Likewise, much work has been done to characterize carboxysomes—compartments in which CO_2_ is concentrated to enhance carboxylation in photosynthetic bacteria–and adapt them for engineering purposes^44–46^. The modularity of carboxysomes and other CO_2_ concentrating mechanism components facilitates reconstitution in other organisms^47,48^. An active area of research is reconstitution in plants^49–51^, as part of a broad strategy to increase plant yields^52–55^. Engineering microbial metabolism with CO_2_ as the primary carbon source would allow the production of sustainably produced biofuels and other high value products^56,57^ and carboxysomes could enhance such strategies. More generally, carboxysomes have been suggested as a modular method for partitioning non-native pathways from native metabolism^22^. However, we have strong indication that performance of CO_2_ concentrating mechanisms will depend on how encapsulation interplays with transporters or other exogenous conditions setting the supply of CO_2_^58,59^, and further systems analysis is required to realize the benefits of carboxysome-based encapsulation^53,60^.

Bacterial microcompartment organelles are not the only organization solution available to the metabolic engineer; scaffolds based on protein, lipid, DNA, and RNA have all shown promise in improving heterologous pathway performance. Each of these strategies have been shown to be effective for the enhancement of heterologous biosynthesis in various contexts^37,61–65^, possibly due to an increase in the local concentration of the intermediate species^66^, as well as potentially due to local effects on enzyme performance^67^. These studies organized diverse biosyntheses, including of mevalonate, resveratrol, 1,2-propanediol, and molecular hydrogen, suggesting that many different enzymatic pathways could be enhanced by scaffolding.

Having developed tools to control the localization of heterologous biosynthetic pathways to these organizing structures, crucial questions remain: what pathways are suitable for organization? And what benefits might be accrued by organizing pathways in one way versus another? A variety of numerical simulation frameworks exist capable of simulating the spatial-temporal concentrations of metabolites in cells^68^. Some modeling efforts have described the potential for kinetic enhancement by scaffolds^69,70^. There has also been analytic and computational analysis of optimal compartment size and distribution of enzymes between multiple compartments^71^. Here, we will explicitly compare organelle-like to scaffold-based organization to better understand the potential trade-offs between the two approaches. Our goal is not to rebuild the reaction-diffusion simulation framework or perform numerical investigations of a particular system, but to provide parametric insight into the design criteria across cellular organization strategies.

We will argue that organization in microcompartment organelles can confer benefits commensurate to substantial enzyme kinetic improvements, and that the choice of organization strategy is impacted both by intrinsic pathway properties and extrinsic culture conditions. All of these factors are important to consider in choosing the appropriate organization strategy for a pathway of interest, and here we demonstrate how the intrinsic and extrinsic factors define the design space of the trade-offs between strategies.

## Results

### Pathway encapsulation in bacterial microcompartments can provide benefits comparable to protein engineering

We will outline the potential metabolic engineering benefits that could be derived from pathway organization using two different strategies: encapsulation in the Pdu microcompartment of *Salmonella* and other enteric bacteria, and organization using a scaffold (which could be organized by means of protein, lipid, or nucleic acid) (Fig. 1C). To begin, we will examine the benefits of encapsulation and scaffolding for two pathways.

To address the kinetic consequences of encapsulation in Pdu microcompartments, we make use of a model^43^ developed to analyze the native function of the microcompartment organelles. A key result of this analysis is that microcompartments significantly enhance pathway flux for a range of external substrate concentrations. We predict that the natively encapsulated system enjoys a four-order of magnitude enhancement in flux upon encapsulation, as compared to free diffusion of the enzymes in the cytosol^43^. Appropriately chosen heterologous pathways might also accrue such flux enhancements, as well as potentially reduce the loss of pathway intermediates to the extracellular space.

To model a scaffolded system, we simply assume that the enzymes in question are localized to a volume equivalent to that occupied by the Pdu microcompartments, but without a diffusion barrier. The velocity of transport between the scaffold volume and the cytosol is set to that of free diffusion. The model formulation we use here is agnostic to the underlying scaffolding platform (protein, lipid, or nucleic acid) or its microscopic organization, as we assume a well-mixed scaffold volume.

**Figure 2:**
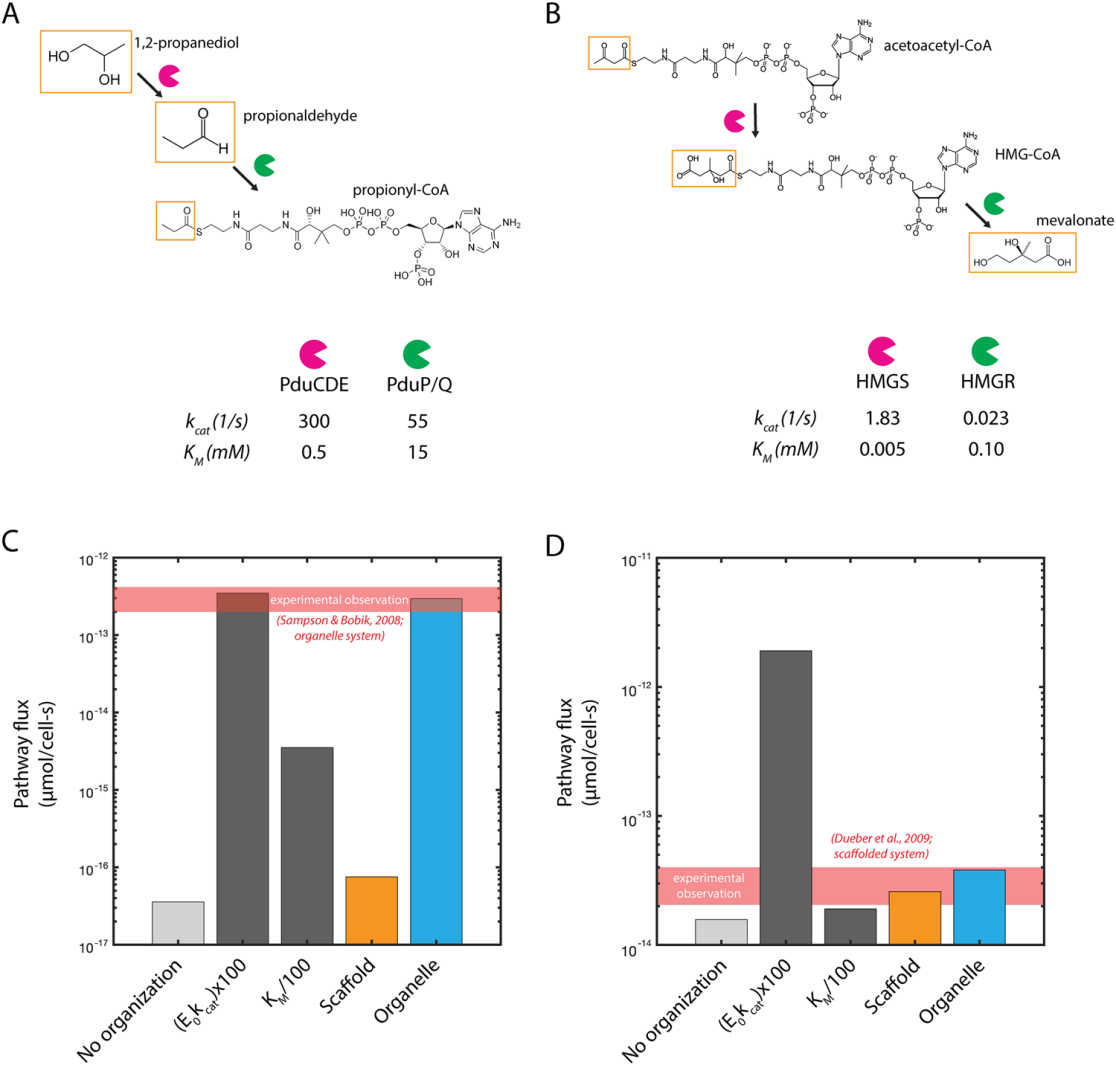
Relevant substrates, intermediates, products, and enzyme kinetic parameters for (**A**) native Pdu microcompartment metabolism and (**B**) mevalonate biosynthesis. Predicted pathway flux for (**C**) native Pdu microcompartment metabolism and (**D**) mevalonate biosynthesis for native kinetics without organization; 100-fold improvement of *k*_*cat*_ for both pathway enzymes; 100-fold improvement in *K*_*M*_ for both pathway enzymes; native kinetics with organization on a scaffold; and native kinetics with organization in a microcompartment organelle. The predictions here are based on an external substrate concentration of 50 mM 1,2-propanediol, as is typically used in experiments^42^. We use the same external substrate concentration (50 mM) in the mevalonate case. Experimental observations in (**C**) and (**D**) are calculated from *S. enterica*^42^ and from titer measurements for a scaffolded system from^63^.

We analyze the consequences of organization using these two strategies for two model biochemical processes: native Pdu microcompartment metabolism and the heterologous mevalonate biosynthetic pathway. The compounds and enzyme kinetic parameters for each pathway are in Fig. 2A,B. We adapt the modeling approach used for the native Pdu system to make flux predictions for the heterologous mevalonate pathway by adjusting the enzymatic kinetic parameters, cell membrane permeability to metabolites, and enzyme abundance and stoichiometry. While the first substrate of the mevalonate pathway (acetoacetyl-CoA) is produced intracellularly, rather than entering from the extracellular space (in the case of 1,2-PD), we approximate the generation of acetoacetyl-CoA upstream as a constant extracellular concentration in the context of our kinetic model. This approximation could correspond to production of acetoacetyl-CoA by a relatively fast and reversible upstream enzyme, or to more complex homeostatic control of the acetoacetyl-CoA concentration in the cytosol, both resulting in an effectively constant concentration of acetoacetyl-CoA far from the organelle or scaffold. To evaluate consistency between our results and this assumption, we confirm that the concentration gradient in the cytosol is small across a wide range of external substrate concentrations for all the organizational cases we tested (Fig. S1). The initial external concentration parameters represent conditions that have been used in prior experiments, and thus provide a benchmark against which to compare the estimates of pathway flux from our model.

We first compare the effects of organization to traditional metabolic and enzyme engineering strategies (such as improvements to the *k*_*cat*_ or *K*_*M*_ kinetic parameters). We are interested in whether metabolic engineering of spatial organization is worth the additional effort. Here, we use the native Pdu microcompartment pathway as an example. If, for instance, engineering efforts increased the *k*_*cat*_ of each of the two key enzymatic steps 100-fold, or decrease the *K*_*M*_ of each key step 100-fold, the improvement in flux would be as shown in Fig. 2C,D (as compared to the native system with no organization). Improvements of this magnitude for both enzymes represent a significant technical challenge and would be non-trivial to achieve for an arbitrary enzymatic system.

We find that encapsulation of native Pdu metabolism in an organelle is practically as effective as large improvements in *k*_*cat*_, and more effective than large improvements in *K*_*M*_, with respect to increasing the total flux through the pathway (Fig. 2C). See the Methods for a detailed description of this calculation and the model in general. The calculated concentrations of each metabolite for each kinetic case are shown in Fig. S2. The dramatic improvement in flux upon encapsulation is due to a large increase in the intermediate concentration in the vicinity of the second pathway enzyme, exceeding the saturating concentration. Moreover, this benefit comes without a significant increase in the cytosolic concentration of this intermediate (Fig. S2). Our comparisons indicate that the native Pdu microcompartment metabolic pathway benefits substantially more from encapsulation than it would from scaffolding (Fig. 2C).

In contrast with the dramatic benefit of enhancement from encapsulation in the Pdu system, the pathway to produce mevalonate accrues similar flux enhancement from an organelle- or scaffold-based organization strategy (Figs 2D; S3). Our analysis is consistent with the experimental observation that organizing the mevalonate pathway on a protein scaffold increased titers^63^. We expect marginal additional benefit from an encapsulation approach in this case, since the Michaelis-Menten constants *K*_*M*_ for the second enzyme is small, whereas we calculate that the potential for flux enhancement by encapsulation in an organelle is high for pathways kinetically similar to native Pdu metabolism (that is, with larger *K*_*M*_) (Fig. 2C,D).

In both pathways, increased flux can also be achieved by increasing the *k*_*cat*_ of each enzymatic step, but the improved flux comes at the cost of greatly increased loss of intermediate species to the extracellular space (or to other cellular process, in the case that there are competing reactions in the cytosol) (Fig. S4). This tradeoff between pathway throughput and intermediate loss may be important to consider in cases where the intermediate species is toxic or easily escapes across the cell membrane. In such cases, the protein engineering strategy may be less appealing than organization, despite similar predicted flux enhancement.

These kinds of calculations can be made for any enzymatic pathway for which the kinetic parameters are known (or can be approximated). Already, they provide a design rule for the bioengineer: pathways with relatively high *K*_*m*_ for the second reaction in a sequence will benefit from encapsulation, while those with relatively low *K*_*m*_ may do just as well with co-localization through scaffolding. Nonetheless, these calculations offer a relatively restricted view of the system, addressing performance for particular pathways under particular culture conditions. We are interested in the performance and robustness of each strategy when applied to different pathways or varying external conditions. The next section addresses these issues by mapping out the range of parameters, or design-space, for which encapsulation or scaffolding would be preferred.

### Optimal organization strategies for biosynthetic pathways differ based on pathway properties and culture conditions

The benefits of encapsulation versus scaffolding can depend on intrinsic factors, such as pathway kinetics (discussed in the prior section) or the cell membrane permeability to the metabolites, as well as extrinsic factors, such as the external concentration of substrate. We examine tradeoffs between flux enhancement and intermediate loss for both intrinsic and extrinsic factors. We initially analyze the effects of varying external substrate concentration independently.

**Figure 3:**
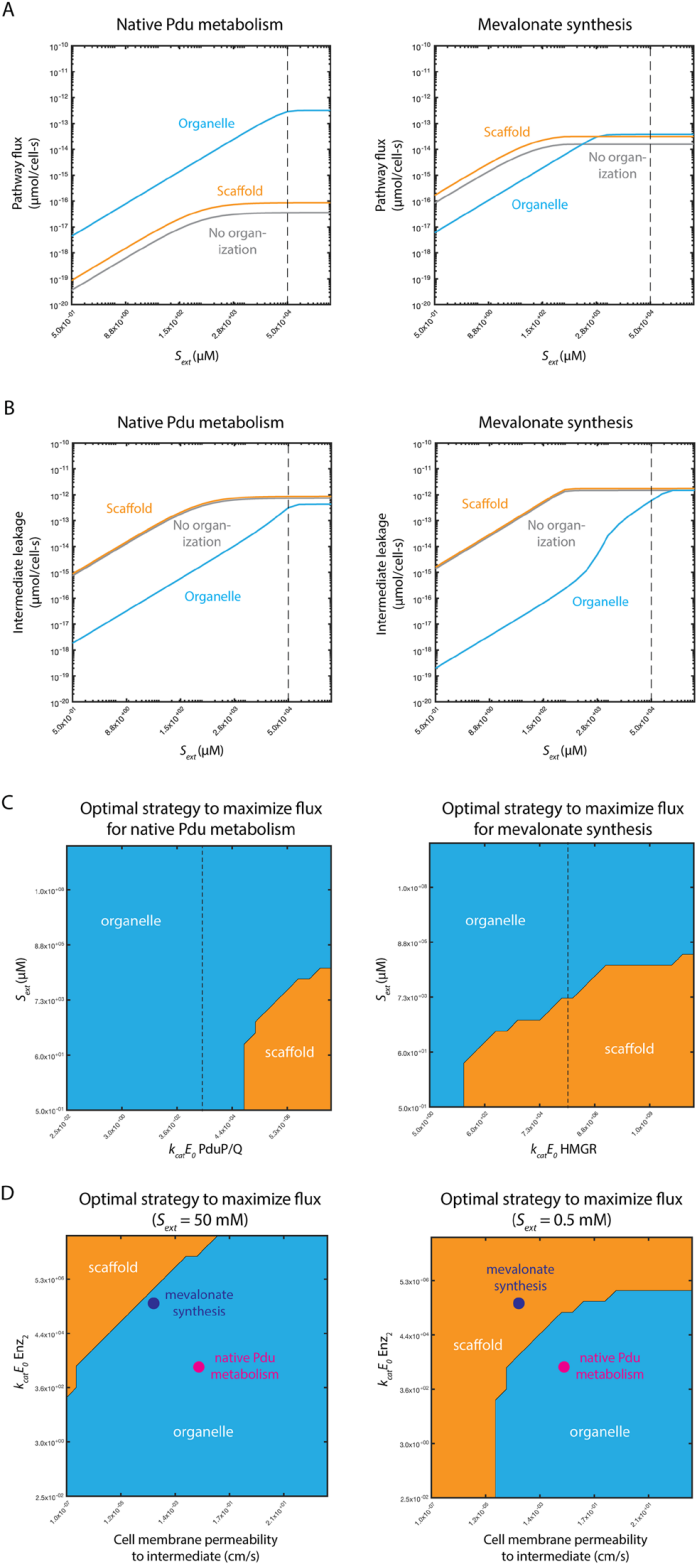
(**A**) Predicted flux for (left) native Pdu microcompartment metabolism and (right) mevalonate biosynthesis without organization (grey); with organization on a scaffold (orange); and with organization in an organelle (blue) as a function of external substrate concentration *S*_*ext*_. (**B**) Predicted intermediate leakage for (left) native Pdu microcompartment metabolism and (right) mevalonate biosynthesis without organization (grey); with organization on a scaffold (orange); and with organization in an organelle (blue) as a function of external substrate concentration *S*_*ext*_. (**C**) Predicted optimal organizational strategy for (left) native Pdu microcompartment metabolism and (right) mevalonate biosynthesis as a function of external substrate concentration *S*_*ext*_ and the abundance and kinetics of the second pathway enzyme *k*_*cat*_*E*_0_ (PduP/Q and HMGR, respectively). Baseline parameter values are shown with a black dashed line. (**D**) Predicted optimal organizational strategy for native Pdu microcompartment metabolism (magenta) and mevalonate biosynthesis (purple) as a function of the abundance and kinetics of the second pathway enzyme *k*_*cat*_*E*_0_ (PduP/Q and HMGR, respectively) and the cell membrane permeability to the intermediate at (left) external substrate concentration *S*_*ext*_ = 50 mM and (right) *S*_*ext*_ = 0.5 mM. Regions of parameter space are colored by the optimal organization strategy in that region: organelle (blue); scaffold (orange); or no organization (grey).

For the Pdu system the organelle provides higher flux for all substrate concentrations. However, at lower external substrate concentrations, organizing mevalonate synthesis on scaffolds results in higher flux than localizing it in organelles (Fig. 3A). The organelle strategy supports less flux because lower substrate concentrations result in smaller diffusive gradients into the organelle, reducing the rate of entry of substrate into the organelle. The permeability of the organelle, which would be optimal at higher substrate concentrations, is too restrictive at low substrate concentrations and limits flux. The rate of leakage of intermediate to the extracellular space is also affected; in each case, a scaffold leads to the greatest intermediate leakage, and this disadvantage worsens at low bulk substrate concentration for both pathways (Fig. 3B). This underscores the importance of considering the pathway in question and the desired outcome (flux enhancement or leakage prevention) when selecting organization strategies.

We next consider the effects of one cell-intrinsic property (the abundance and kinetics of the second pathway enzyme) and one cell-extrinsic property (external substrate concentration) simultaneously (Fig. 3C). These design spaces show the optimal organizational strategy to maximize flux as a function of both variables, with the optimal strategy indicated by the color at that parameter value combination. For reference, the dashed line in each panel of Fig. 3C indicates the *k*_*cat*_*E*_0_ value used to construct the one-dimensional responses with respect to *S*_*ext*_ in Fig. 3A. While the topology of these landscapes is similar for both systems (and indeed for any irreversible two-enzyme pair governed by Michaelis-Menten kinetics), there are important quantitative differences. Critically, for the *k*_*cat*_*E*_0_ value of the second enzyme in the mevalonate synthesis pathway (dashed vertical line), we predict that organelle-type organization is favored at high *S*_*ext*_ but scaffolding is favored at low *S*_*ext*_ values (Fig. 3C). A batch-type reactor, therefore, might transition from organelles to scaffolds being optimal during a production run; laboratory-scale pilot experiments are most often conducted in this mode, potentially convoluting different optimality regimes. The same is true for fed-batch processes; this mode of operation is common in industrial- as well as laboratory-scale fermentation. This observation holds for *k*_*cat*_*E*_0_ values several orders of magnitude smaller or larger than our estimate. The same is not true for the native Pdu MCP system, in which organelles are favored for all *S*_*ext*_ values given our assumptions regarding *k*_*cat*_*E*_0_ of PduP/Q (Fig. 3C). This kind of information is key in designing optimally productive biosynthetic processes, and might call for a dynamic organizational transition as culture conditions change, or simply for the choice of an organizational strategy that is robust to the perturbations in question^72^.

We next demonstrate how the optimal organizational strategy changes as a function of two intrinsic pathway properties (rather than one intrinsic and one extrinsic property, as in Fig. 3C). While there are many parameters that can change between pathways, we focus on two key differences between the Pdu MCP system and mevalonate pathway. The values we estimate for the cell membrane permeability to the intermediate and *k*_*cat*_*E*_0_ for the second enzyme differ by approximately two orders of magnitude between the two systems. These permeability estimates are based on the size and charge of the intermediate (see Methods). We therefore calculate the optimal organizational strategy as a function of these two parameters, and indicate the location of each enzyme system in this design space (Fig. 3D). We set all model parameters besides cell membrane permeability and *k*_*cat*_*E*_0_ to the baseline values for the Pdu MCP system. The design space does not qualitatively change if we instead set all the other parameters to those representative of the mevalonate biosynthetic pathway (Fig. S5). We construct the design space for two external substrate concentrations *S*_*ext*_, 50 mM and 0.5 mM. Crucially, the change in external concentration shifts the boundary between the regions in which scaffold and organelle strategies are optimal. At the lower *S*_*ext*_, mevalonate biosynthesis favors a scaffold over an organelle, while organelle organization is still favored for the Pdu MCP.

### Organization can buffer pathways against cell cycle-dependent perturbations

In addition to extrinsic perturbations, such as changes in the external substrate concentration, microbial factories also undergo periodic changes in cell volume due to growth and division. One might also imagine that the size of the scaffold or organelle itself could also vary. To assess the robustness of organized systems to such variation, we examined the performance of organization strategies with respect to changes in both the cell radius *R*_*b*_ and the organizing volume radius *R*_*c*_.

We find that flux through pathways localized inside organelles is unaffected by changing cell size, while flux through the scaffold-organized and unorganized systems are affected by changing cell size (Fig. S6). This observation held both for Pdu metabolism and mevalonate biosynthesis. Erecting a diffusive barrier around the encapsulated system insulates the enclosed chemical microenvironment from changes in cell size as might occur throughout growth, and likely reflects a general function of organelles across kingdoms^73,74^.

In contrast, we find that the flux through encapsulated systems does depend on the size of the organelle (Fig. S6), whereas there is little dependence of flux on scaffold volume size. Furthermore, the apparent optimum in *R*_c_ is intimately linked to the permeability of the microcompartment shell *k*_*c*_. Decreases in *R*_c_ without accompanying increases in *k*_*c*_ unduly limit substrate influx, whereas increased *R*_c_ without decreased *k*_*c*_ compromises the organelle’s intermediate-trapping function (Fig. S7). This may in part explain the relative uniformity in organelle size within a given microcompartment system^29,34,75,76^.

### Enzyme stoichiometry and kinetics, as well as design goals, influence optimal organization strategy

**Figure 4:**
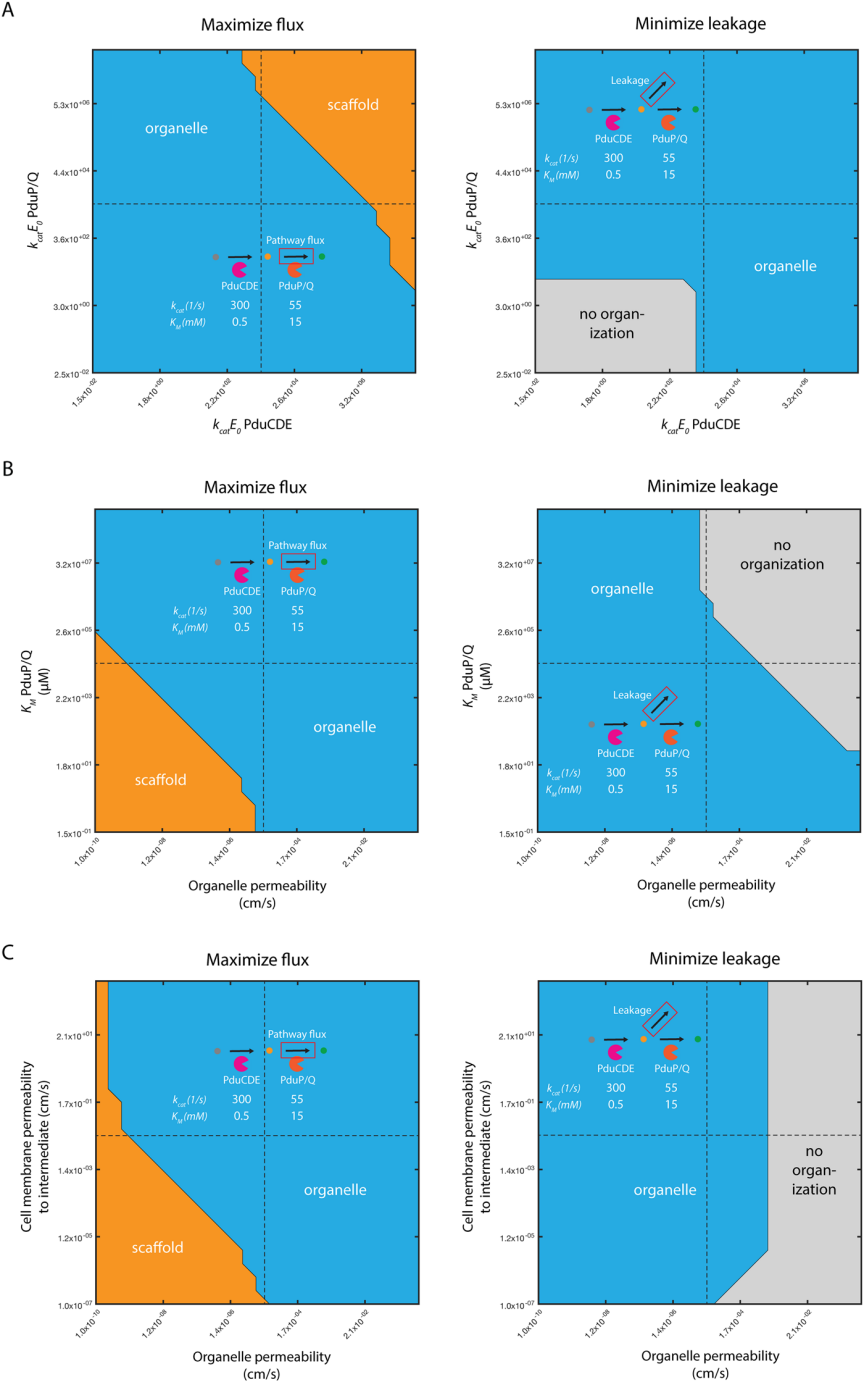
Recommended organization strategy resulting in (left) maximum pathway flux or (right) minimum intermediate leakage for native Pdu metabolism as a function of (**A**) *k*_*cat*_*E*_0_ of PduCDE and PduP/Q, (**B**) organelle permeability and *K*_*M*_ of PduP/Q, and (**C**) organelle permeability and cell membrane permeability. Regions of parameter space are colored by the optimal organization strategy in that region: organelle (blue); scaffold (orange); or no organization (grey). Baseline parameter values are shown with a black dashed line.

We can address the question of organization choice for pathways more generally by examining the relative performance of three strategies (free cytosolic localization of enzymes; scaffolds; and microcompartments) across a wide range of enzyme kinetic parameters. Considering, for instance, the two-enzyme pathway of native Pdu metabolism, we can calculate the optimal strategy as we vary the activity (*k*_*cat*_*E*_0_) of each enzyme (Fig. 4A). This variation can represent either the Pdu enzymes or a different enzymatic pathway. In this example, microcompartment organization is favored with respect to maximizing pathway flux unless the respective *k*_*cat*_*E*_0_ parameters for both enzymes are sufficiently large to render the effect of concentrating intermediate species in the microcompartment insignificant, in which case a scaffold is recommended. Conversely, for small *k*_*cat*_*E*_0_ of both enzymes, a strategy without spatial organization is indicated to minimize intermediate loss (Fig. 4A). These design space predictions of organization performance can be made for arbitrary organizational strategies and metabolic pathways, given appropriate kinetic models.

### The chemical character of the substrate and intermediate also have an impact on organization choice

In addition to the kinetic properties of the pathway enzymes, we can consider the effect of different substrates and intermediates on the choice of appropriate organizational strategies. Once again we compute the recommended organization strategy for the native Pdu metabolic pathway, and vary the values of the relevant model parameters. In this case, several parameters could be affected by the chemical character of the species, including transport across the cell membrane and transport in and out of the microcompartment organelle. If, for instance, transport of the substrate and intermediate across the microcompartment shell is slow, scaffold expression may be favored if the *K*_*M*_ of the second enzyme is sufficiently low (Fig. 4B). On the other hand, if escape of the intermediate across the organelle boundary is slow, scaffolding may be favored regardless of cell membrane permeability (Fig. 4C), since the cell itself can perform the organelle’s intermediate-concentrating function in this case. It may be possible to optimize the permeability of the microcompartment shell to the kinetics of the desired pathway and broaden the range of conditions under which an organelle is the optimal pathway^41,77^. All of these factors must be considered when choosing an appropriate organization strategy. This is particularly important when comparing biosynthetic pathways with substrates and intermediates of different sizes, which might reasonably be expected to have disparate transport properties at the cell membrane and microcompartment shell.

### Enzyme mechanism can alter the potential benefits of organization strategies

In the above examples, we consider irreversible, Menten-Michaelis kinetics for each enzymatic step of each pathway. This assumption holds for the Pdu microcompartment case, but not for all systems. For example, in the carboxysome, a carbon-fixation organelle of cyanobacteria, a key enzymatic step catalyzing the interconversion of CO_2_/HCO_3_^−^ is reversible, limiting the benefit of organelles to concentrate intermediate species^58^. Selective permeability of the carboxysome does not result in increased CO_2_ concentration^59^. The reversibility of the CO_2_/HCO_3_^−^ conversion imposes a fundamental limit on the concentration of CO_2_ that can be achieved in the organelle (Fig. S8A); on the other hand, selective permeability combined with enzyme irreversibility allows the development of a very high local intermediate concentration if the intermediate is selectively trapped (Fig. S8B; purple line). The comparison between the reversible and irreversible kinetic models highlights the need to account for detailed aspects of kinetic mechanism, such as cofactors, inhibition, and other dynamic effects. Recent studies have also indicated that the local chemical environment of nucleic acid-based scaffolds can have beneficial effects on enzyme kinetics^78^; detailed kinetic effects of this kind can be incorporated into future models as they are elucidated.

## Discussion

### A decision framework for organizational choice

Above, we outline the potential for the spatial organization of heterologous pathways to greatly enhance their performance. This approach compares well with traditional enzyme engineering approaches. Furthermore, we describe a general systems parameter analysis to guide the choice of appropriate organizational strategies for metabolic engineering. Several key parameters must be accounted for: (I) enzyme kinetics; (II) substrate and intermediate chemical properties; and (III) external culture conditions. Some of these properties, such as the external substrate concentration and the transport properties of the cell membrane, can influence the supply of substrate to the pathway, while others, such as the presence or absence of competing reactions and the transport properties of the organelle boundary, can influence the loss of metabolic flux to off-target species. We analyze the performance of different organization strategies, and present example organizational recommendations. Moreover, this modeling approach suggests key experiments (*e.g*. variations of external substrate concentration) to probe discrepancies in the performance of different organization strategies. The MATLAB code used to generate the graphics in this manuscript is freely available on GitHub (https://github.com/cjakobson/pduMCPmodel), and we encourage members of the metabolic engineering and synthetic biology communities to explore the organizational performance landscapes for their own systems of interest. We also welcome suggestions of other useful kinetic or organizational regimes to include in future versions of the analysis.

Design spaces of the kind presented here can be constructed for any pair of parameters. They provide a means to survey the organizational performance landscape comprehensively across many orders of magnitude of possible parameter values. This is particularly important when some parameters are not well constrained. The parameters to explore could include those susceptible to manipulation via culture conditions, such as *S*_*ext*_; those that can in principle be engineered, such as *k*_*cat*_*E*_0_; and those that are intrinsic to the relevant biomolecules, such as the cell membrane permeability to the intermediate species. By constructing these design spaces for a variety of parameters, the metabolic engineer can gain a quantitative understanding of which parameters are critical in determining the optimal organization strategy for a given pathway, and can weigh the ease of altering a given parameter against the potential rewards in terms of engineering goals like pathway flux.

### Avenues to improve understanding and prediction of optimal spatial organization

The comparisons we outline above can provide important insights into the choice of optimal organizational strategies for heterologous pathways, but several important aspects of pathway organization remain unexplored. These include product export, cell morphology, competitive reactions, and the detailed organization of the organelles or scaffolds within the host cell. The organization of organelles within cells has been investigated in the context of a constant cytosolic metabolite concentration^78^, and future efforts could combine this and our approaches.

As implemented, our model can incorporate extensions to explore some of these areas, but some questions, notably the effect of competitive cellular reactions and reactions upstream of the organized process, will require the integration of our model with network-wide metabolic models of host processes. Exploring the effect of the detailed subcellular localization of the organizing structures themselves will likewise require modifications to our current model.

### Practical engineering considerations

Here, we evaluate two design goals: the pathway flux and intermediate leakage predicted for each potential organization strategy. We do not attempt to quantify a cost associated with the difficulty of implementing each strategy. We could account for more complex objectives in future by creating composite objective functions for the energetic cost of flux enhancement and leakage, or for the cost of the enzymes and organizing structures themselves^79^, and optimizing across these different factors simultaneously.

These objective functions would be important if, for certain pathways, scaffolding proves challenging to implement due to incompatibilities between the requisite protein tags and the enzymes in question, or that the pores of microcompartment shells are fundamentally of low permeability to the substrates of other pathways. Practical experience with these issues will continue to inform the choice of appropriate strategies, and could eventually be quantified in objective functions. Certain key experiments, such as assays to quantitatively determine the permeability of protein shells to small molecules, will greatly enhance the predictability of engineering pathways in microcompartments.

### Closing thoughts

Organizing biochemistry in both time and space holds tremendous potential to deliver the promise of synthetic biology: the ability to produce medically and industrially important molecules at high yield and high titer with minimal environmental disruption. Spatial organization of the kind we discuss is but one of many important approaches; techniques to use multiple (or no) cells, to detect and transport metabolites, and to exert dynamic control on short time scales are of critical importance, as well. We posit that parametric studies to map the design spaces of each of these approaches will be key in optimizing biosyntheses.

## Materials and Methods

### Contact for resource and reagent sharing

For questions and further information regarding software and models used herein, please contact NMM (niallmm@gmail.com).

### Method details

#### Model

The reaction-diffusion model used herein is substantially the same as that described in Jakobson *et al*., PLoS Computational Biology, 2017, in which we explored the native function of the Pdu MCP system in *S. enterica*. The analytical and numerical approach is described in detail in that manuscript, but the important assumptions can be summarized as follows:

1. We assume a spherically symmetrical organelle or scaffold at the center of a spherically symmetrical cell.
2. We consider the system at steady-state.
3. We assume that the external concentrations of the substrate (*S*_*ext*_) and intermediate are constant.
4. We assume that the activity of the enzymes can be described by irreversible Michaelis-Menten kinetics.

The governing equations are as follows in the cytosol and in the organelle or scaffold-like structure:

Cytosol:

$${\rm{D}}{\nabla }^{2}{S}_{1}({\rm{r}})=0$$

$${\rm{D}}{\nabla }^{2}{S}_{2}({\rm{r}})=0$$

Organelle/scaffold:

$${\rm{D}}{\nabla }^{2}{{\rm{S}}}_{1}({\rm{r}})-{{\rm{R}}}_{1}=0$$

$${\rm{D}}{\nabla }^{2}{{\rm{S}}}_{2}({\rm{r}})+{{\rm{R}}}_{1}-{{\rm{R}}}_{2}=0$$

where

$${{\rm{R}}}_{1}=\frac{{{\rm{V}}}_{1}{{\rm{S}}}_{1}({\rm{r}})}{{{\rm{K}}}_{1}+{{\rm{S}}}_{1}({\rm{r}})}$$

and

$${{\rm{R}}}_{2}=\frac{{{\rm{V}}}_{2}{{\rm{S}}}_{2}({\rm{r}})}{{{\rm{K}}}_{2}+{{\rm{S}}}_{2}({\rm{r}})}$$

The boundary conditions at the cell membrane are:

$${\rm{D}}\frac{d{{\rm{S}}}_{1}}{dr}({\rm{r}}={{\rm{R}}}_{{\rm{b}}})={{\rm{k}}}_{m}^{S1}({{\rm{S}}}_{1,{\rm{out}}}-{{\rm{S}}}_{1}({\rm{r}}={{\rm{R}}}_{{\rm{b}}}))$$

$${\rm{D}}\frac{d{{\rm{S}}}_{2}}{dr}({\rm{r}}={{\rm{R}}}_{{\rm{b}}})={{\rm{k}}}_{m}^{S2}({{\rm{S}}}_{2,{\rm{out}}}-{{\rm{S}}}_{2}({\rm{r}}={{\rm{R}}}_{{\rm{b}}}))$$

Likewise at the scaffold or organelle boundary:

$${\rm{D}}\frac{d{{\rm{S}}}_{1}}{dr}({\rm{r}}={{\rm{R}}}_{{\rm{c}}})={{\rm{k}}}_{c}^{S1}({{\rm{S}}}_{1}({\rm{r}}={{\rm{R}}}_{{\rm{c}}})-{{\rm{S}}}_{1,organelle})$$

$${\rm{D}}\frac{d{{\rm{S}}}_{2}}{dr}({\rm{r}}={{\rm{R}}}_{{\rm{c}}})={{\rm{k}}}_{c}^{S2}({{\rm{S}}}_{2}({\rm{r}}={{\rm{R}}}_{{\rm{c}}})-{{\rm{S}}}_{2,organelle})$$

And by symmetry at the cell center in the case of no organization:

$$\frac{d{{\rm{S}}}_{1}}{dr}({\rm{r}}=0)=0$$

$$\frac{d{{\rm{S}}}_{2}}{dr}({\rm{r}}=0)=0$$

In the case of organelle- or scaffold-based organization, a closed-form analytical solution to the governing equations can be found, provided we assume that the metabolite concentration inside the organelle or scaffold region is constant. This solution is used to generate the various figures comparing organization strategies; see^43^ for the derivation and complete analytical solutions. A scaffold-like behavior is created by setting the permeability at the organelle boundary to approximate free diffusion (that is, k_c_^S1^ = k_c_^S2^ ~ 10^3^). In the case with no organization, we instead use a numerical solution, as the analytical solution does not hold in this regime. The numerical solution at steady state is generated by a finite difference routine implemented in MATLAB; again see^43^ for more details on the governing equations and boundary conditions used in the numerical routine.

#### Model parameters

Enzyme kinetic parameters were available from existing literature, as were estimates of cell size, organelle size, enzyme copy number, and the diffusivity of molecules in the cytoplasm. References for these values are detailed in the tables below. We made *a priori* estimates of the cell membrane permeabilities according to the methods of Robinson^80^. The microcompartment shell permeability was set to optimize native pathway flux as described for our prior model^43^.

The following table summarizes the important model parameters for the Pdu MCP system:

**Table:** 

**Parameter**	**Meaning**	**Estimated Value**	**Units**
k_c_^S1^	Permeability of the Pdu MCP to propionaldehyde	10^−5^	cm/s
k_c_^S2^	Permeability of the Pdu MCP to 1,2-PD	10^−5^	cm/s
R_b_	Radius of the bacterial cell	5 × 10^−5^	cm
R_c_	Radius of the Pdu MCP	10^−5^ ^81^	cm
D	Diffusivity of metabolites in the cellular milieu	10^−5^ ^82^	cm^2^/s
k_m_^S1^	Permeability of the cell membrane to propionaldehyde	10^−2^ ^80^	cm/s
k_m_^S2^	Permeability of the cell membrane to 1,2-PD	10^−2^ ^80^	cm/s
k_catCDE_	Maximum reaction rate of a PduCDE active site	3 × 10^2^ ^83^	1/s
N_CDE_	Number of PduCDE enzymes per cell	1.5 × 10^3^ ^81^	Per cell
K_MCDE_	Michaelis-Menten constant of PduCDE	5 × 10^2^ ^83^	μM
k_catPQ_	Maximum reaction rate of a PduP/Q active site	55^84^	1/s
K_MPQ_	Michaelis-Menten constant of PduP/Q	1.5 × 10^4^ ^84^	μM
N_PQ_	Number of PduP/Q enzymes per cell	2.5 × 10^3^ ^81^	Per cell
S_ext_	External 1,2-PD concentration	5.5 × 10^4^ ^42^	μM
A_out_	External propionaldehyde concentration	0^42^	μM

In the case of mevalonate synthesis, the parameters are altered as follows:

**Table:** 

**Parameter**	**Meaning**	**Estimated Value**	**Units**
k_m_^S1^	Permeability of the cell membrane to acetoacetyl-CoA	10^−4^	cm/s
k_m_^S2^	Permeability of the cell membrane to HMG-CoA	10^−4^	cm/s
k_catCDE_	Maximum reaction rate of a HMGS active site	1.83^85^	1/s
N_CDE_	Number of HMGS enzymes per cell	5 × 10^5^ ^63^	Per cell
K_MCDE_	Michaelis-Menten constant of HMGS	5^86^	μM
k_catPQ_	Maximum reaction rate of a HMGR active site	0.023^87^	1/s
K_MPQ_	Michaelis-Menten constant of HMGR	1 × 10^2^ ^87^	μM
N_PQ_	Number of HMGR enzymes per cell	5 × 10^5^ ^63^	Per cell

#### Converting literature observations to flux predictions

For the systems we examine here, previous literature has described either cellular growth on 1,2-PD as the sole carbon source^42^ or the volumetric titer of mevalonate in a scaffolded system^63^. To compare with our model, which generates predictions on a molecules-per-cell basis, we must convert these experimental observations to a comparable measurement.

For the native Pdu microcompartments, Sampson and Bobik observe a doubling time of approximately 5–10 hours during exponential phase growth on 1,2-PD as the sole carbon source (see Figure 3 of^42^). Assuming that a bacterial cell has a mass of approximately 0.3 pg and that half of the flux through the microcompartment pathway (by mass) can be used for cell growth, the Sampson growth observation predicts a steady-state flux of approximately 3 × 10^−13^ μmol/cell-s.

In the case of mevalonate biosynthesis by a scaffolded system, Dueber and colleagues report a titer of approximately 10 mM mevalonate after 2 days of culture, after which time the concentration changes little (see Figure 5 of^63^). Assuming constant production over this time and a cell density of 2 OD ~ 2 × 10^9^ cells/mL, this titer corresponds to a steady-state flux of approximately 3 × 10^−14^ μmol/cell-s.

### Quantification and statistical analysis

MATLAB R2016b (MathWorks) was used for all computation and to generate graphical representations of the results.

### Data and software availability

The model used herein is freely available on GitHub (https://github.com/cjakobson/pduMCPmodel) under a GNU General Public License.

## Electronic supplementary material

Supplementary Information
