# Supplementary material for: Spatially organizing biochemistry: choosing a strategy to translate synthetic biology to the factory
Source: Sci Rep. 2018 May 29;8:8196. doi: 10.1038/s41598-018-26399-0 (PMC5974357; doi:10.1038/s41598-018-26399-0)
Supplement: Supplementary file 1 — Supplementary Information [file 41598_2018_26399_MOESM1_ESM.pdf]

Supplemental Information

To accompany: Spatially organizing biochemistry: choosing a strategy to translate synthetic biology to the factory

Christopher M. Jakobson, Danielle Tullman-Ercek, Niall M. Mangan

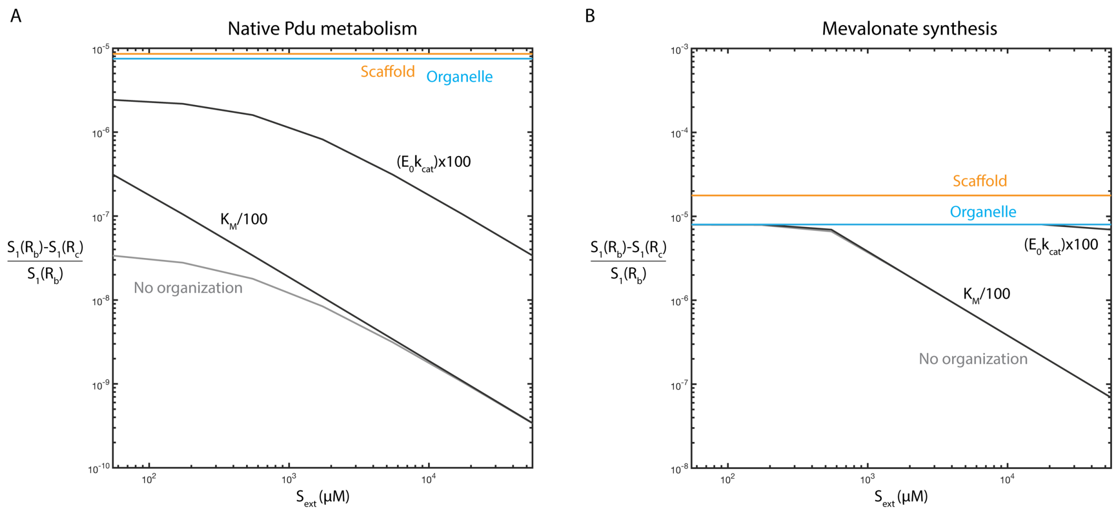

Figure S1. Concentration gradient across the cytosol, defined as the relative change in the concentration of S_1 from the outer edge (R_b) to the inner edge (R_c) of the cytosol, for (A) native Pdu metabolism and (B) mevalonate synthesis for each of five organization cases we consider (see Fig. 2CD) as a function of external S_1 concentration (S_{ext}). In no case does the relative gradient exceed 10^{-4} for any S_{ext}.

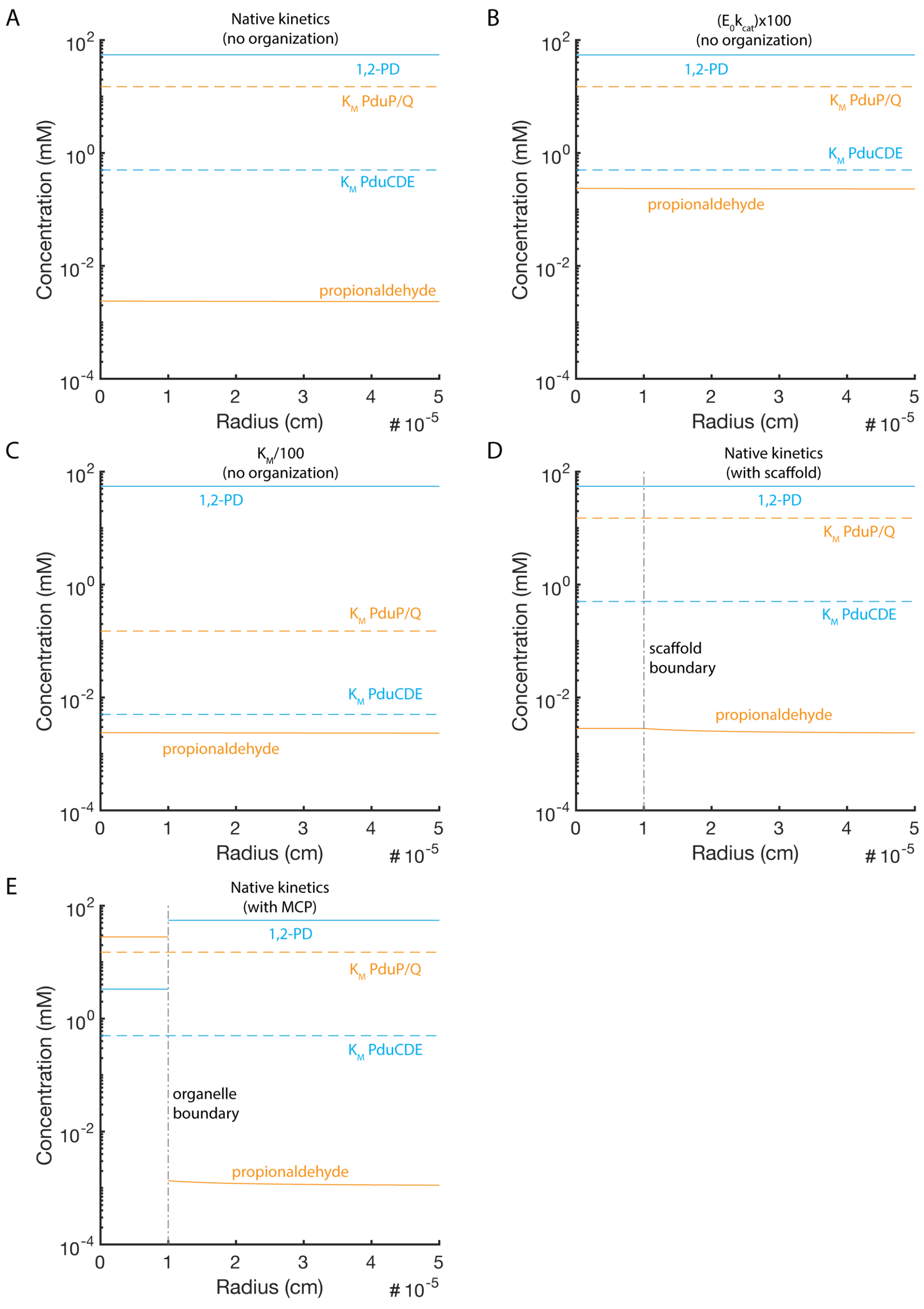

Figure S2. Concentration profiles as a function of cell radius for native Pdu metabolism assuming (A) native kinetics without organization; (B) 100-fold improvement of k_{cat} for both pathway enzymes; (C) 100-fold improvement in K_M for both pathway enzymes; (D) native kinetics with organization on a scaffold; and (E) native kinetics with organization

in a microcompartment organelle. 1,2-propanediol (blue) and propionaldehyde (orange) concentrations are shown in solid lines; K_M values for PduCDE (blue) and PduP/Q (orange) are shown in dashed lines. The organelle boundary is shown in a black dashed line where relevant.

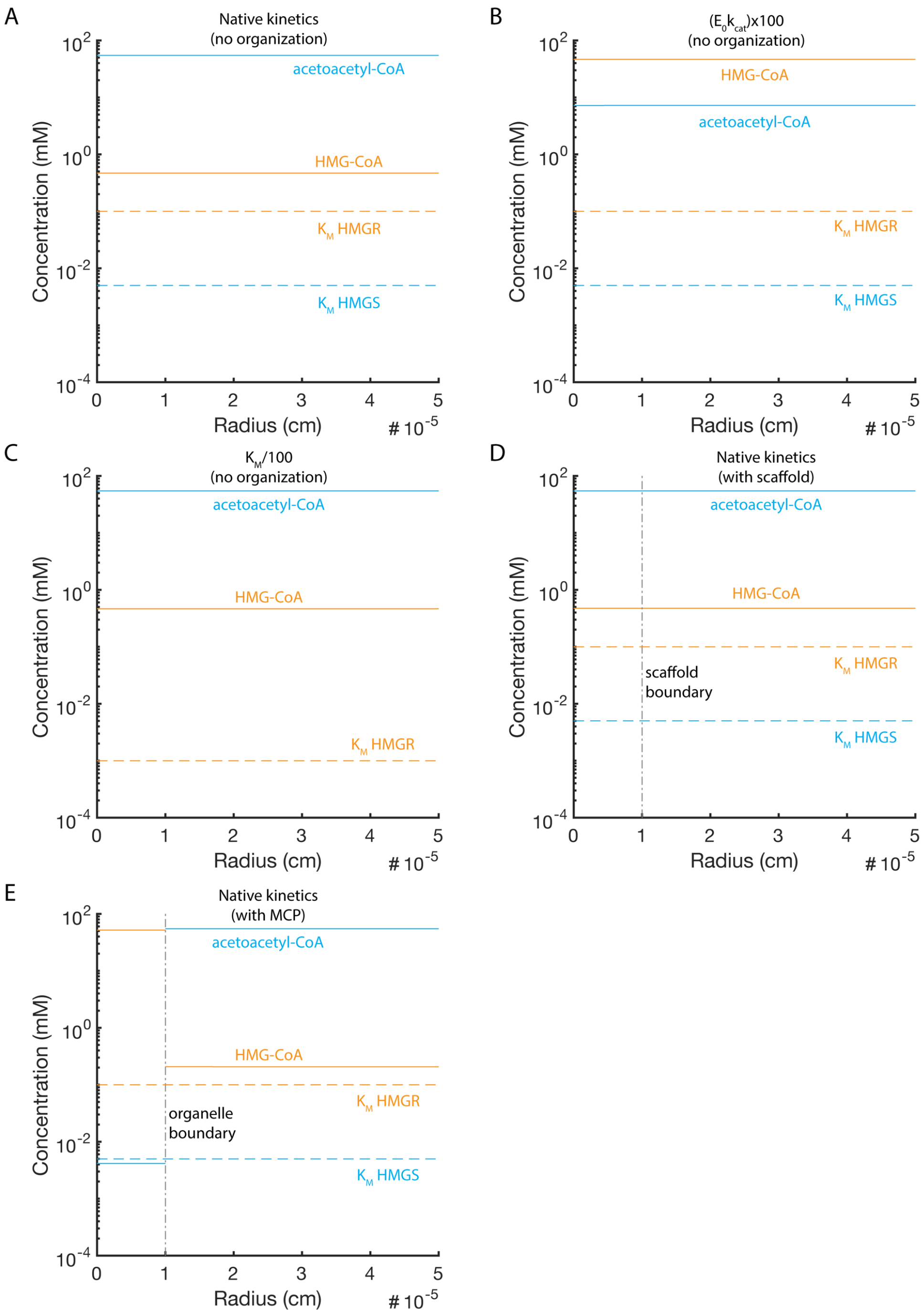

Figure S3. Concentration profiles as a function of cell radius for mevalonate synthesis assuming (A) native kinetics without organization; (B) 100-fold improvement of k_{cat} for both pathway enzymes; (C) 100-fold improvement in K_M for both pathway enzymes; (D) native kinetics with organization on a scaffold; and (E) native kinetics with organization

in a microcompartment organelle. Acetoacetyl-CoA (blue) and HMG-CoA (orange) concentrations are shown in solid lines; K_M values for HMGS (blue) and HMGR (orange) are shown in dashed lines. The organelle boundary is shown in a black dashed line where relevant.

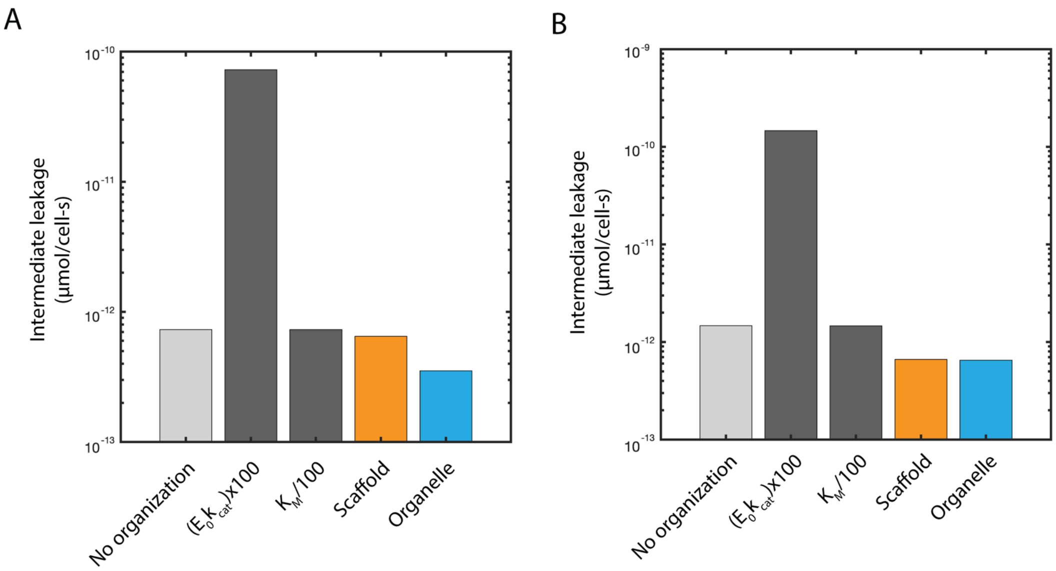

Figure S4. Predicted rate of intermediate leakage into the extracellular space for (A) native Pdu microcompartment metabolism and (B) mevalonate biosynthesis for native kinetics without organization; 100-fold improvement of k_{cat} for both pathway enzymes; 100-fold improvement in K_M for both pathway enzymes; native kinetics with organization on a scaffold; and native kinetics with organization in a microcompartment organelle. The predictions here are based on an external substrate concentration of 50 mM 1,2-propanediol, as is typically used in experiments (Sampson and Bobik, 2008).

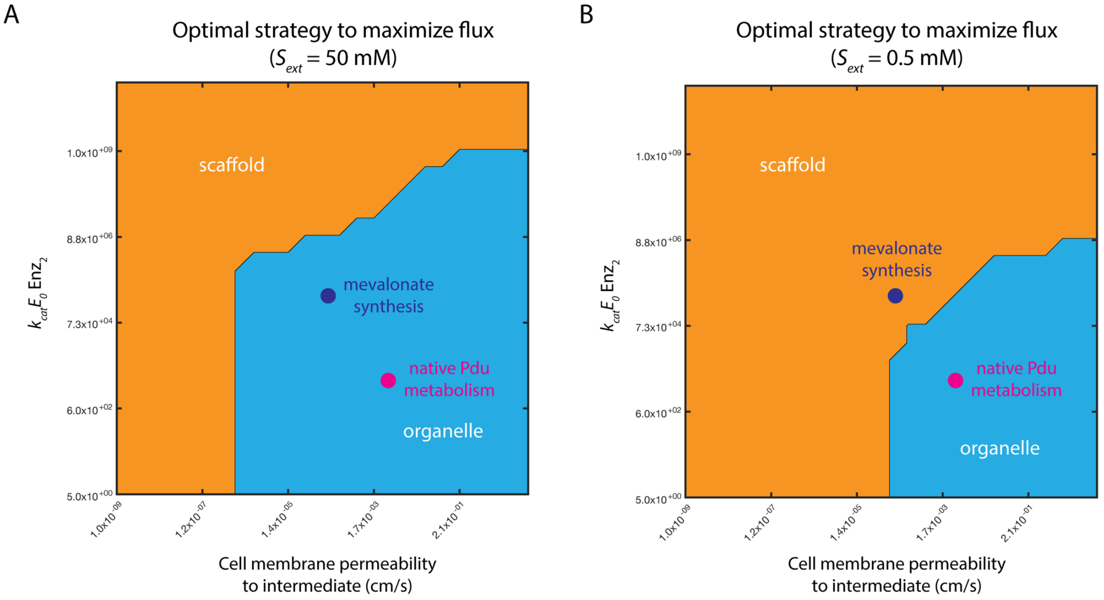

Figure S5. Predicted optimal organizational strategy for native Pdu microcompartment metabolism (magenta) and mevalonate biosynthesis (purple) as a function of the abundance and kinetics of the second pathway enzyme $k_{cat}E_0$ (PduP/Q and HMGR, respectively) and the cell membrane permeability to the intermediate at (A) external substrate concentration S_{ext} = 50 mM and (B) S_{ext} = 0.5 mM. Regions of parameter space are colored by the optimal organization strategy in that region: organelle (blue); scaffold (orange); or no organization (grey). The analysis is identical to that of Fig. 3F, except that the baseline model parameters (excluding those varied in the phase space) are set to those of the mevalonate biosynthetic pathway, not the Pdu MCP system.

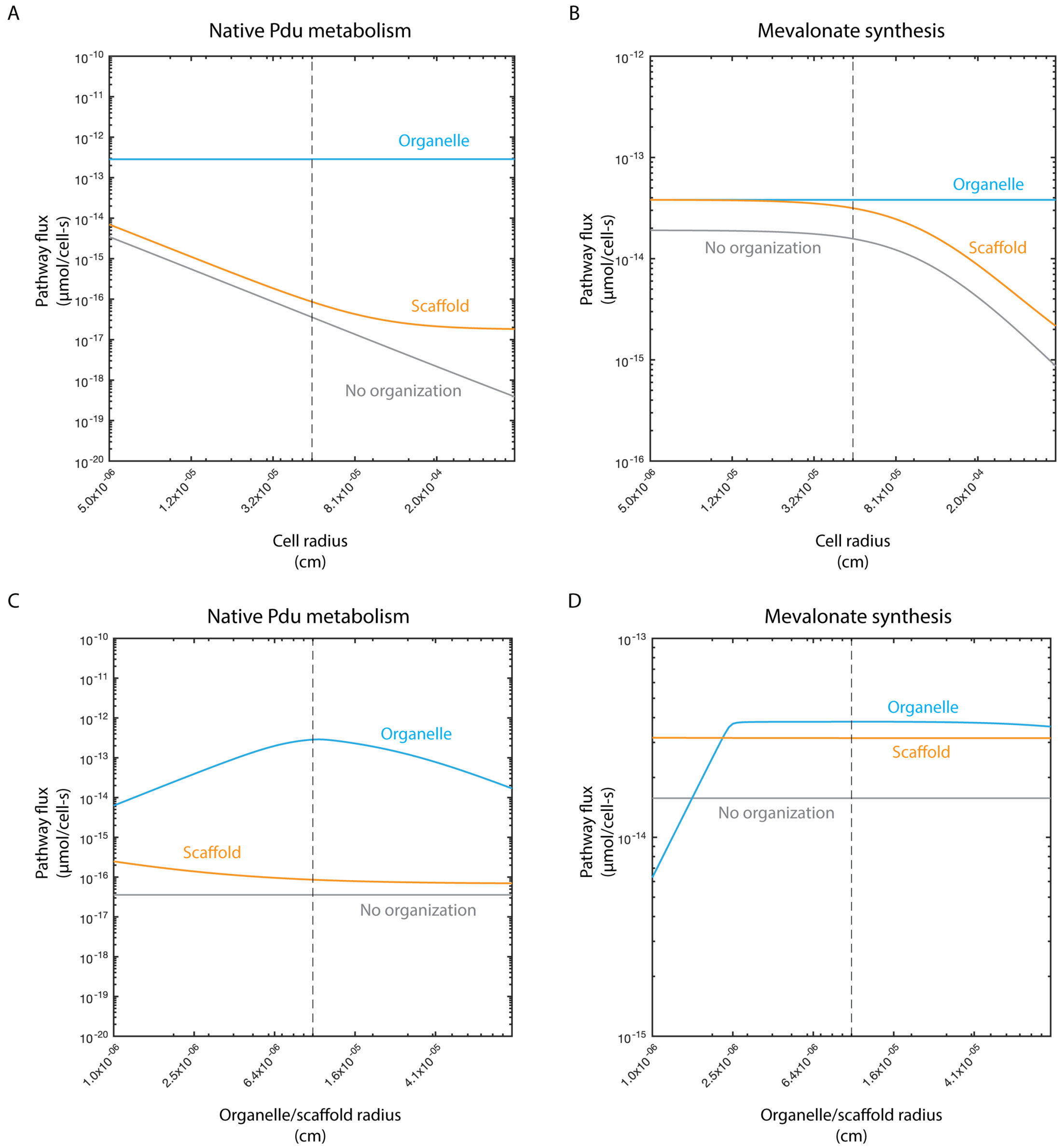

Figure S6. Predicted flux for (A) native Pdu microcompartment metabolism and (B) mevalonate biosynthesis without organization (grey); with organization on a scaffold (orange); and with organization in an organelle (blue) as a function of cell radius R_b. Predicted flux for (C) native Pdu microcompartment metabolism and (D) mevalonate biosynthesis without organization (grey); with organization on a scaffold (orange); and

with organization in an organelle (blue) as a function of organelle/scaffold radius R_c. Baseline parameter values are shown with a black dashed line.

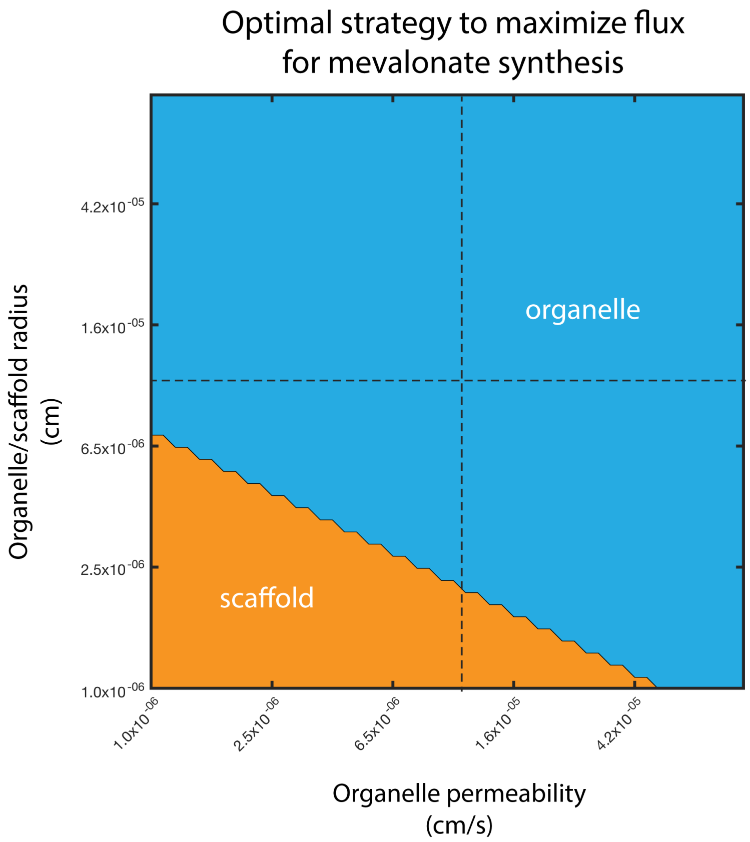

Figure S7. Recommended organization strategy resulting in maximum pathway flux for mevalonate biosynthesis as a function of organelle permeability and organelle/scaffold radius. Regions of parameter space are colored by the optimal organization strategy in that region: organelle (blue); scaffold (orange); or no organization (grey). Baseline parameter values are shown with a black dashed line.

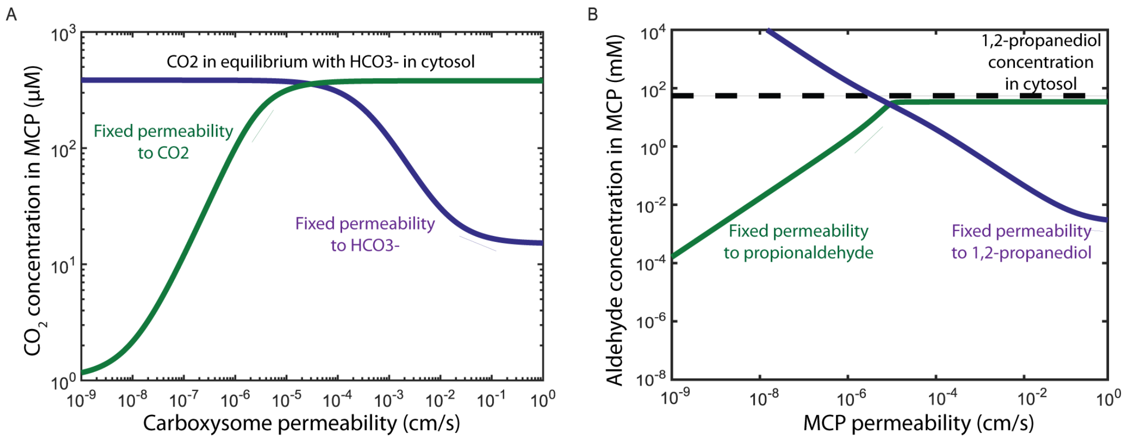

Figure S8. Intermediate concentration in (A) the carboxysome and (B) the Pdu microcompartment as a function of (A) carboxysome permeability and (B) microcompartment permeability. Intermediate concentration is shown in (A) with fixed permeability to CO_2 (green) and fixed permeability to HCO_3^- (purple) and in (B) with fixed permeability to propionaldehyde (green) and fixed permeability to 1,2-propanediol (purple). Reproduced from Jakobson et al., 2017; Mangan and Brenner, 2014.
